# The Role of Food Consumption in the Global Syndemic: A Scoping Review and Conceptual Model

**DOI:** 10.3390/ijerph22060897

**Published:** 2025-06-05

**Authors:** Giovanna Garrido, Fernanda Costa Severo, Samantha Marques Vasconcelos Bonfim, Laís Ferreira Dias, Ana Luiza Gomes Domingos, Andrew D. Jones, Antonio Mauro Saraiva, Dirce Maria Lobo Marchioni, Eliseu Verly Junior, Evandro Marcos Saidel Ribeiro, Olivier Jolliet, Flavia Mori Sarti, Aline Martins de Carvalho

**Affiliations:** 1 Department of Nutrition, School of Public Health, University of São Paulo—FSP-USP, São Paulo 01246-904, SP, Brazil; giovannagarrido@usp.br (G.G.); fernandacostasevero@usp.br (F.C.S.); samantha.mbonfim@usp.br (S.M.V.B.); laisfdias@usp.br (L.F.D.); marchioni@usp.br (D.M.L.M.); 2 School of Arts, Sciences and Humanities, University of São Paulo—EACH-USP, São Paulo 03828-000, SP, Brazil; algdomingos@gmail.com (A.L.G.D.); flamori@usp.br (F.M.S.); 3 Department of Nutritional Sciences, School of Public Health, University of Michigan, 1415 Washington Heights, Ann Arbor, MI 48109-2029, USA; jonesand@umich.edu; 4 Center for Artificial Intelligence—C4AI, University of São Paulo, Av. Prof. Lúcio Martins Rodrigues, 370, Butantã, São Paulo 05508-020, SP, Brazil; saraiva@usp.br; 5 Institute of Advanced Studies, Planetary Health Brazil Network, University of São Paulo—IEA-USP, Rua da Praça do Relógio, 109, São Paulo 05508-050, SP, Brazil; 6 Department of Epidemiology, Institute of Social Medicine, State University of Rio de Janeiro (UERJ), 10 Maracanã, Rio de Janeiro 20550-900, RJ, Brazil; verlyjr@ims.uerj.br; 7 School of Economics, Business Administration and Accounting, University of São Paulo, Av. dos Bandeirantes, 3900, Ribeirão Preto 14040-902, SP, Brazil; esaidel@usp.br; 8 Department of Environmental and Resource Engineering, Technical University of Denmark, 2800 Kongens Lyngby, Denmark; ojoll@dtu.dk

**Keywords:** climate change, food consumption, malnutrition, obesity, undernutrition

## Abstract

The increase in chronic diseases and climate change in recent decades has been driven by food systems that affect both human health and the environment. This study investigated the interrelation between food consumption, obesity, undernutrition, and climate change, aiming to understand how these factors connect within the global syndemic. The methodology used was a scoping review, in which 12 articles were analyzed after an initial search that resulted in 11,208 references. The references were screened using the Rayyan software (Rayyan Systems Inc. (Doha, Qatar), version 1.6.1 and web-based version), removing duplicates and assessing the studies based on eligibility criteria. The articles addressed different aspects, such as the relationship between food consumption, obesity, undernutrition, and climate change, providing data on how food insecurity and socioeconomic conditions influence these conditions. In sequence, we developed a conceptual model to offer a detailed view of the factors affecting the global syndemic, considering the availability of food, its accessibility, stability in supply, and its use in the diet. The model recognizes that climate change affects food consumption both directly and indirectly. Direct effects include the impact of extreme weather events—such as floods and droughts—on the availability, access, quantity, and quality of food. Indirectly, climate change exacerbates socioeconomic vulnerabilities and disrupts food systems in more structural ways, contributing to increased food insecurity. The findings revealed that food insecurity, in turn, can lead to both obesity and undernutrition, particularly among vulnerable populations. There was a scarcity of studies that integrated the relationship between undernutrition, climate change, and food consumption, especially in certain regional contexts such as Latin America. The evidence gathered in the literature and the conceptual model provide a foundation for future research and the development of more effective public policies that integrate food issues, public health, and climate change in a more holistic and interconnected approach.

## 1. Introduction

In recent decades, we have witnessed the progressive growth of chronic disease rates in nearly all regions of the world. This growth occurs in parallel with challenges related to climate change, with food systems being one of the major drivers of this phenomenon [1]. Food systems encompass all actors and activities involved in the production, processing, distribution, consumption, and disposal of food. These systems are made up of subsystems that influence each other and interact with other systems, demonstrating a complex network of interactions [2]—which is related to the global syndemic of obesity, undernutrition, and climate change [3]—capable of generating various impacts on human and environmental health.

The concept of syndemic, introduced by Merrill Singer [4], emphasizes how diseases cluster and interact under the influence of adverse social conditions [4]. The Global Syndemic, as elaborated by [3], extends this framework to include the synergistic effects of non-communicable diseases and environmental degradation—highlighting the need for systemic, not siloed, solutions. The word syndemic refers to the synergistic interaction of multiple epidemics or health problems within a specific population, driven by shared social, environmental, and economic factors. In the case of the Global Syndemic, it refers to a set of interconnected pandemics—obesity, undernutrition, and climate change—that reinforce each other, creating a burden on healthcare systems and global resources. They are considered pandemics because of their geographic spread and the scale of their impact on health and natural systems. Rather than addressing these issues in isolation, the Global Syndemic proposes integrated solutions that take into account the interactions and the urgency of addressing these issues collectively [3].

According to the World Obesity Federation (2024) [5], more than 1 billion people globally are living with obesity—a figure projected to surpass 1.5 billion by 2035 if no significant interventions occur. Obesity is a major risk factor for cardiovascular diseases and cancer, which are the leading causes of death worldwide, especially in developing countries [5]. Simultaneously, the 2024 SOFI report estimated that 735 million people experienced hunger in 2023, while over 2.4 billion faced moderate or severe food insecurity. This condition encompasses insufficient food intake in terms of both quality and quantity, and can be reflected in indicators such as stunting, underweight, and micronutrient deficiencies [6]. Finally, completing the triad of the global syndemic, climate change is associated with rising average temperatures, changes in humidity patterns (wetter or drier, depending on the region), and an increased frequency of extreme environmental phenomena. These changes can directly affect both the quantity and the nutritional quality of agricultural production, which relies on a dynamic balance of appropriate resources. Consequently, the nutritional quality of food consumed is directly influenced by climate change [3].

Studies indicate that rising atmospheric CO_2_ concentrations can reduce the levels of essential nutrients in staple foods such as rice, wheat, and maize, thereby compromising the nutritional quality of global diets [6]. In addition, elevated CO_2_ has been associated with decreased protein content in plant tissues. Considering that protein is one of the three primary macronutrients—along with carbohydrates and lipids—it becomes imperative to quantify its presence when estimating the impact of altered plant quality on human nutrition in a CO_2_-enriched world [7], particularly in light of the broader implications for global food security and the nutritional adequacy of diets, especially among vulnerable populations.

Extreme weather events, such as droughts and floods, also directly affect agricultural production, resulting in shortages of nutritious foods and increased reliance on products with low nutritional value, which exacerbates food insecurity, especially in vulnerable regions [8]. Recent modeling studies have shown consistently negative effects of rising atmospheric CO_2_ on the availability of protein, iron, and zinc by 2050 across all regions of the world, although the magnitude of these effects varies between countries [9].

Common factors to these three pandemics of obesity, undernutrition, and climate change include: changes in land use for food production and the forms of food production; urban design to facilitate the flow of produced foods and allow individuals access, both physically and financially; consumer food behavior, regarding the financial capacity linked to income and access to food, as well as food preparation techniques for consumption. Analyzing the dynamics between these factors is essential to clarify the effects of potential interventions, as the syndemic is a consequence of deeper systemic problems that require actions at the individual, institutional, and political levels [3].

Although studies on the global syndemic require a systemic approach due to the complexity of the topic, some of the current research presents this issue isolated, referring only to obesity [10], undernutrition [11], or climate change [12], and in a linear way, relating the variables directly [13]. To address this, a scoping review was conducted to systematically describe existing research that deals with at least two components of the syndemic and identify knowledge gaps. The results of the articles included in the review and insights from experts were aggregated to create a conceptual model, attributing complexity to the relationships. Given the central role of food consumption in mediating both undernutrition and obesity, as well as its environmental impacts, this review aims to map the current evidence on how dietary patterns and food behaviors contribute to—and can potentially mitigate—the Global Syndemic.

## 2. Materials and Methods

### 2.1. Scope Review, Research Question, and Eligibility Criteria

Given the complexity of the food system and its repercussions on the determinants of food consumption in the global syndemic, this study adopted the method proposed by the Joanna Briggs Institute (JBI) for scope reviews [14] The recommendations for the development of the Preferred Reporting Items for Systematic Reviews and Meta-Analysis Protocols—Extension for Scoping Reviews (PRISMA-ScR) were followed, as well as its adaptation for presenting the results obtained during the stages of this review [15].

The formulation of the question for this review considered the strategy that encompasses population, concept, and context (PCC) for inclusion: (a) regarding the population: studies that address the general population, without age and/or sex limitations; (b) regarding the concept: specific aspects of food consumption that may include types of diet, eating habits, food availability, food access, etc.; and (c) regarding the context: different settings, including both national and international contexts.

The following inclusion criteria were applied: documents that provided the full text, written in English, Portuguese, or Spanish, with the latter two languages adopted to target studies focusing on South America; documents where food consumption was related to at least two of the three components of the global syndemic (undernutrition, obesity, and climate change) published in the last five years (2018 to 2024).

The combination of food consumption terms and the other two components of the global syndemic was determined because the scientific literature has already consolidated the relationship between food consumption and the isolated components of the global syndemic [3]. In this regard, we sought documents that addressed the relationship between these components to identify potential gaps in the literature.

To ensure a comprehensive review, the following documents and resources were excluded from the analysis based on specific criteria: (1) Studies that related food consumption to only one component of the global syndemic, as the review aimed to explore the interconnected nature of the syndemic. (2) Articles that addressed food consumption in isolation, without considering its relationship to the broader context of health, the global syndemic, or other related factors. (3) Studies that discussed components of the global syndemic without relating them to food consumption. (4) Publications that focused solely on enzymes, nutrients, food types, or diets without a broader connection to the global syndemic. (5) Animal studies, as the review focused on human populations. (6) Reviews, systematic reviews, and literature reviews, as the aim was to include only original research articles. (7) Technical research reports, commercial publications, white papers, catalogs, datasheets, application notes, user guides, and quickstart guides, as they are not peer-reviewed resources. (8) Book chapters, as they typically do not meet the criteria for peer-reviewed articles. (9) Research protocols, which present planned methodologies without results. (10) Studies on eating disorders, snacking, night eating, sleep disorders, and pre/postprandial examinations, as they did not align with the review’s focus on the global syndemic and food consumption. (11) Studies with specific sample populations (e.g., bariatric surgery patients or pregnant women), as the review aimed to include broader, generalizable findings on food consumption patterns.

### 2.2. Literature Review

Searches were conducted in the electronic databases Web of Science—Core Collection (Clarivate Analytics), SCOPUS (Elsevier), and MEDLINE/PubMed (via National Library of Medicine).

The search strategies were designed to combine at least one association term, one food consumption term, and at least two components of the global syndemic (Supplementary Material Table S1). It is important to note that truncation was applied, meaning the use of an asterisk (*) in the root of words to find terms from the same lexical field written in different forms. This approach led to studies being found by employing a combination of descriptors and terms found in the titles, abstracts, and keywords of relevant articles on the topic to form a search strategy in databases (Supplementary Materials Tables S1–S3). The database searches lasted for 10 days and were divided into two phases: PubMed and WOS from 29 January 2024 to 2 February 2024, and Scopus from 15 February 2024 to 16 February 2024. In the interval between these dates, the results were downloaded and uploaded to Rayyan in order to proceed with the next steps listed below:

1. The identified articles were imported into the Rayyan review management software (https://www.rayyan.ai/ accessed on 11 March 2024) (Qatar Computing Research Institute, Doha, Qatar), a free online and mobile app that allows for blinded collaboration among reviewers and improves data screening;
2. In Rayyan, duplicates were removed;
3. Three blinded reviewers assessed the identified articles. First, title screening was performed for all documents. Each document was reviewed by at least two reviewers to ensure consistency. Articles accepted by at least two reviewers were included. In cases of disagreement, a third reviewer was consulted to make the final decision. The next step was abstract screening, which followed the same procedure as the title selection. Each document was classified for inclusion or exclusion based on its title and abstract;
4. A record of the decisions was kept on the platform;
5. The full texts of the included abstracts were retrieved and considered for the review.

After the final step of document inclusion, data extraction was performed in Excel by three independent reviewers. To detail the information, an auxiliary tool developed by the reviewers was used (Supplementary Material Table S4). Information was collected on the name of the first author, the journal in which the material was published, the country of the research sample, the year of publication, the data collection period, the study type, the study objective, and the identification of food consumption variables and global syndemic components. Lastly, the relationships between food consumption and the global syndemic and their respective influencers were extracted.

The protocol was registered under the name “The role of food consumption in the global syndemic: scoping review protocol” and can be accessed via the DOI: 10.17605/OSF.IO/FYKSC.

### 2.3. Development of the Conceptual Model

**Figure 1:**
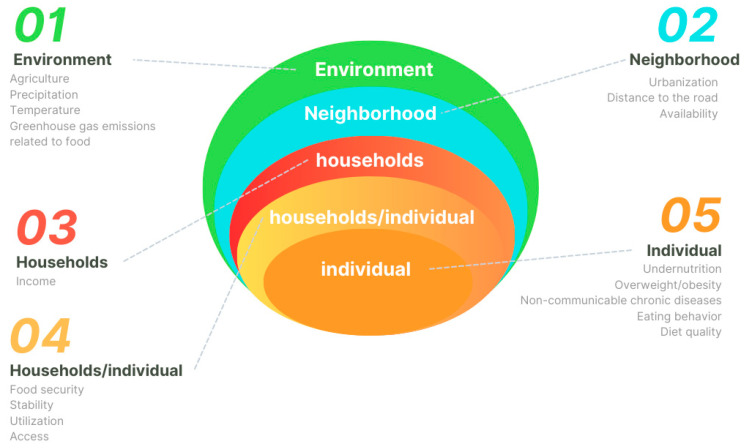
Dimensions of the conceptual model about food consumption and global syndemic. Source: The authors (2025).

Based on the relationships between food consumption and the global syndemic and their respective influencers, a conceptual model was developed with the help of Canva^®^ software (2025). The determinants of food consumption and their interrelations with the global syndemic were organized based on the dimensions of food and nutritional security from the Food and Agriculture Organization (2024) [2], following the socioecological model (Figure 1): individual, household, individual/household, neighborhood, environment, and cross-cutting factors.

From the connections identified in the review, the initial model was refined through a collaborative effort of the authors. This process involved a two-hour hybrid meeting of the research group, composed of experts from various fields such as nutrition and complex systems modeling. During the discussion, a preliminary model was presented that described the relationships between food consumption and the components of the global syndemic. The connections were analyzed individually, with discussions on whether the relationships were appropriate and which ones should be added, removed, or adjusted based on the researchers’ expertise and the study’s objectives.

- Individual: undernutrition, consumption of ultra-processed foods, unhealthy eating patterns, overweight/obesity, and non-communicable chronic diseases;
- Household: income, diet quality, diet diversity, food security, and the double burden of undernutrition;
- Neighborhood: access and nutritional transition;
- Environment: greenhouse gas emissions (GHGE), fauna, temperature, and precipitation.
- Cross-cutting variables: These affect multiple dimensions of the model and were considered throughout the refinement phases. An example is “sex” (limited to the binary biological sex of female and male), which can directly influence food access (individual/household dimension), income (household), and indirectly affect diet quality (individual/household dimension). These variables were highlighted with a specific symbol in the conceptual model, making it easier to identify their broad influence.

This final structure allowed for a better visualization of the relationships between the factors that determine food consumption in the context of the global syndemic.

During the refinement meeting, eight relationships were excluded, twelve relationships were improved, both in classification and connections, and three were approved (Supplementary Material Table S6). An example of an adjustment to the model was the inclusion of the “Climate Change” variable, highlighting that GHG, precipitation, and temperature directly influence this variable, which in turn impacts food consumption only indirectly, through other connections and intermediate steps

The final version of the model presented one additional dimension, as described below:

- Individual: variables related to the individual level, such as BMI and 24-h food recall;
- Individual/Household: variables that encompass both individual and household-level data, such as dietary diversity in the household and food cost;
- Household: variables related to household conditions, such as socioeconomic status;
- Neighborhood: population-related variables, such as urbanization;
- Environment: environmental variables, such as temperature and agriculture;
- Cross-cutting variables: no changes to the definition.

Each dimension was represented by a color code in the conceptual model—orange for individual, a gradient of orange and red for individual/household, red for household, blue for neighborhood, green for environment, and purple for cross-cutting. The system dynamics were verified through connections between the factors, which were illustrated by arrows:

- Continuous: relationships identified in the literature;
- Dashed: relationships proposed by the experts;
- Unidirectional: indicating that the influence occurs in the direction of the arrow;
- Bidirectional: indicating mutual interaction between the factors.

## 3. Results

### 3.1. Findings from the Literature Review

**Figure 2:**
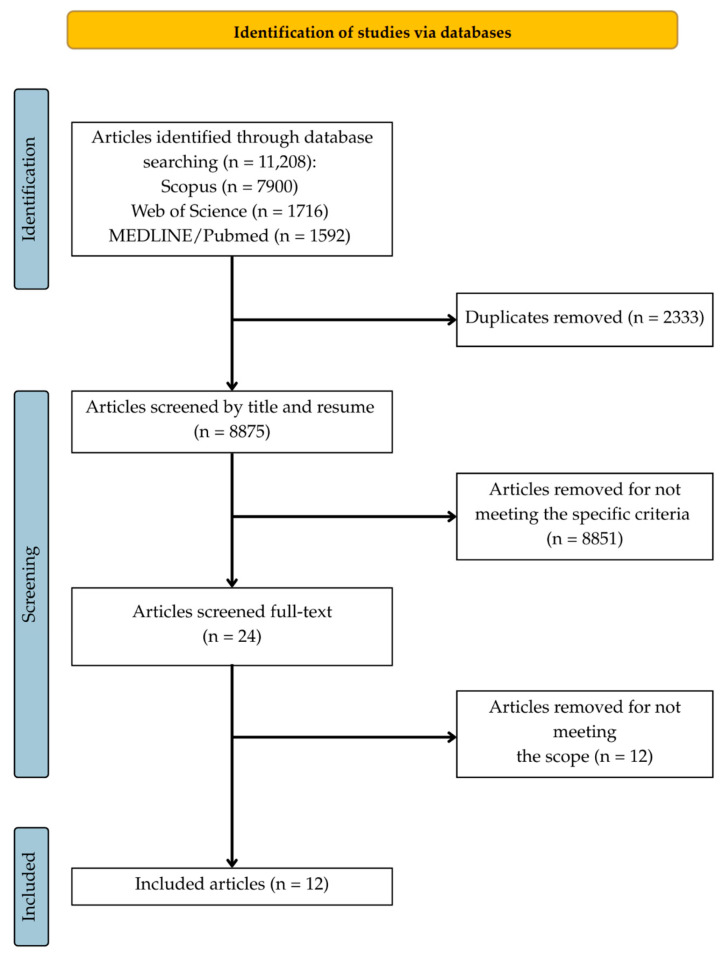
Study selection process. Source: The authors (2025).

A total of 11,208 references were identified through the search. Of these, 2333 duplicates were automatically detected and removed using Rayyan software (Rayyan Systems Inc., version 1.6.1 and web-based version), leaving 8873 records for title and subsequently abstract screening. After applying the inclusion and exclusion criteria (e.g., studies involving pregnant women, COVID-19, animal models, specific diseases, among others), 24 studies were selected for full-text assessment. Of these, 12 were excluded for not meeting the scope of the review, resulting in 12 studies included for data extraction (Figure 2). The included articles—referenced numerically throughout this review—along with their characteristics and extracted data, are available in the Supplementary Materials.

### 3.2. Selected Studies and Data Tabulation

**Figure 3:**
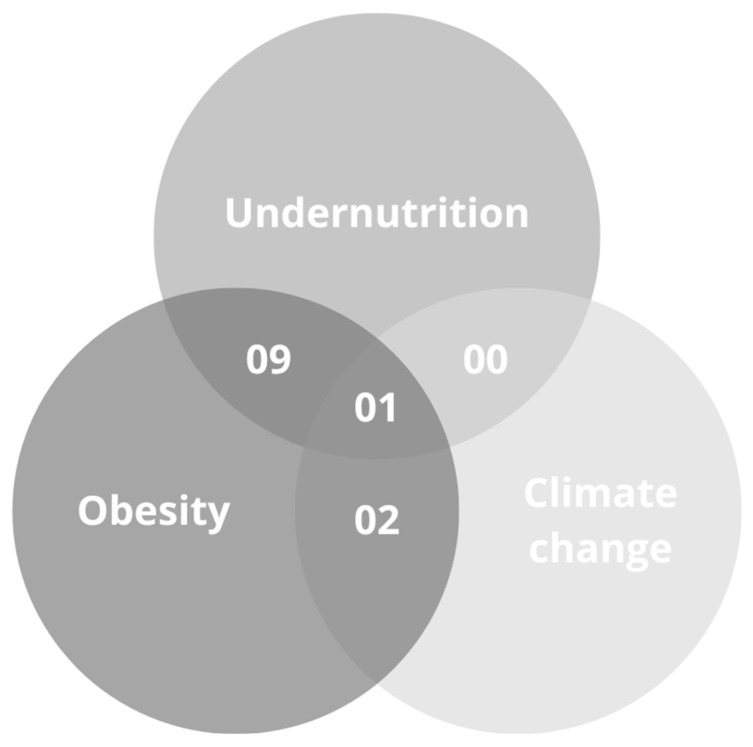
Venn Diagram illustrating the overlapping topics in articles addressing undernutrition, obesity, and climate change. Source: The authors (2025).

Of the 12 articles selected for inclusion, six were published in 2022. The types of articles included the relationship between food consumption, obesity, and undernutrition (n = 9), food consumption, obesity, and climate change (n = 2), and obesity, undernutrition, and climate change (n = 1). No documents were found that pointed to the relationship between food consumption, undernutrition, and climate change. These data can be observed in Figure 3.

After the data extraction step, the articles were listed and numbered to facilitate data and result management and improve visualization (Supplementary Material—Table S7).

In the first phase of organizing the variables, that is, in the general listing of all variables found in the documents, we identified 30 variables with different definitions (subsequently grouped with their similar counterparts) and their various evaluation measures (Supplementary Material—Table S8).

After tabulating the variables, the main relationships observed in the analyzed data were identified and documented (Table 1).

### 3.3. Conceptual Model Development

**Figure 4:**
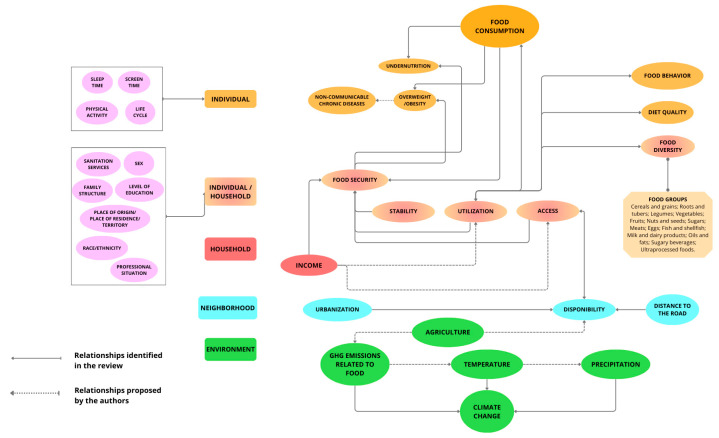
Conceptual model of the relationships between food consumption and global syndemic. Source: The authors (2025). The continuous arrows indicate the relationship proposed by the literature and dashed arrows indicate the relationship proposed by the researchers.

From then on, a conceptual model (Figure 4) was built, enabling a global visualization of the connections found between the variables. It is possible to observe that food consumption directly interacts with individual factors and indirectly with factors from the food environment (household and neighborhood), the environment itself, and other parts of the food systems, such as production. It is widely demonstrated that agricultural exploration influences the amount of greenhouse gases (GHG) emitted, which, as exposed in this review, promote climate change, also driven by extreme changes in temperature and precipitation levels [13,16,17,18,19]. This food production dynamic, in turn, affects the availability of food, which also varies with the urbanization of the area and with the distance to the road [16,18,20,21]. Availability then interferes with the physical and economic access to food, which is bidirectionally related to its utilization (adequate consumption of safe and nutritious foods) and, consequently, food consumption [17,18,19,20,21,22,23,24]. Furthermore, consumption is also directly influenced by eating behavior, diet quality, and dietary diversity [20,21,24,25]. Thus, food consumption impacts undernutrition, food security, and overweight and obesity, the latter being intrinsically related to the occurrence of non-communicable diseases, as demonstrated by extensive literature and reference health organizations [19,24,25,26]. Moreover, food security is affected by stability—defined by the Food and Agriculture Organization (FAO) as the ability to have food consistently over time—which is bidirectionally related to utilization, and by income, and influences the levels of undernutrition, overweight, and obesity. Finally, based on the authors’ experience and discussions with specialists, household income also affects utilization and access.

**Table 1:** Frequency of occurrence of the relationship in the review.

Category	Variables	Observed Relationships	References ^1^	
Food Security and Nutrition	Access, Undernutrition, Food Composition, Food Security	Access → Undernutrition, Food Composition → Quality, Food Security → Obesity	1	[22]
			3	[24]
			5	[18]
Social and Economic Determinants	Income, Employment, Family Structure, Education	Income → Food Security, Employment → Obesity, Family Structure → Undernutrition, Education → Dietary Diversity	4	[23]
			7	[16]
			10	[13]
Environmental and Geographical Influences	Geography, Fauna, Temperature, Precipitation	Geography → Access, Fauna → Undernutrition, Temperature → Undernutrition, Precipitation → Obesity	1	[22]
			5	[18]
Behavioral and Lifestyle Factors	Dietary Behavior, Physical Activity, Sleep, Screen Time	Dietary Behavior → Obesity, Physical Activity → Obesity, Sleep → Undernutrition	1	[22]
			8	[20]

^1^ The references correspond to the numbers presented in Table S7.

## 4. Discussion

The discussion on food systems is essential in light of the urgency of global challenges, particularly in the context of the global syndemic, which links obesity, undernutrition, and climate change. As already stated, it is central to Planetary Health which aims at achieving health and wellbeing for all [27]. In the context of food systems, food consumption is a central element in this debate, reflecting not only nutritional patterns but also socioeconomic inequalities, cultural dynamics, and environmental impacts [28].

### 4.1. Relationships Between Food Consumption and Components of the Global Syndemic

Food consumption, obesity, undernutrition, and climate change are interconnected within the global syndemic, a phenomenon that highlights how socio-economic and environmental factors exacerbate interconnected health issues. In contexts of food insecurity, extreme climate events such as droughts and floods increase the vulnerability of populations, resulting in the coexistence of obesity and undernutrition, especially in regions most affected by climate change. Although obesity and undernutrition may seem like opposing conditions, both share the limitation of access to nutritious foods, which leads to harmful food choices and unsustainable dietary patterns [3,6]. In food insecure situations, the most vulnerable populations face elevated risks of chronic diseases such as diabetes, hypertension, and heart diseases, along with nutritional deficiencies caused by the lack of essential foods [29,30].

The food industries play a central role in this dynamic by promoting ultra-processed foods, which are high in calories, sugar, saturated fats, and sodium, significantly contributing to the global epidemic of obesity and non-communicable chronic diseases [30]. These products, low-cost and highly accessible, are primarily consumed by low-income populations, who face barriers to accessing fresh and healthy foods. Additionally, the production and processing of ultra-processed foods generate a large amount of greenhouse gas emissions, exacerbating climate change while intensifying food insecurity, creating a vicious cycle [30]. This environmental impact of the food industries is further worsened by food shortages caused by climate change, which increases the reliance on ultra-processed food products due to their long shelf life and ease of transportation and storage [3,31]. Public policies must, therefore, adopt an integrated approach to address food insecurity, nutrition, and climate change, aiming to mitigate the adverse effects of the food industries and promote global sustainability and food security [31].

#### 4.1.1. Food Consumption, Obesity, and Undernutrition

The relationship between food consumption, obesity, and undernutrition has been widely discussed in the literature and is recognized as a complex phenomenon. To better understand this interconnection, we selected articles that address these three dimensions simultaneously. Nine studies were analyzed, exploring the connection between food consumption, obesity, and undernutrition.

In research conducted with young people (children and adolescents), Wu et al. (2019) [22] identified a significant relationship between food insecurity and the risk of overweight and obesity, with an Odds Ratio (OR) of 1.46. This means that for each additional level of severity in food insecurity, the likelihood of developing these conditions increases by 46%. This finding highlights the importance of socioeconomic factors, such as food insecurity, in the risk of these diseases, especially in more vulnerable populations [22].

Yang et al. (2022) [20] found that Chinese children and adolescents who met recommendations for movement behaviors and dietary guidelines had a significantly lower risk of being overweight or obese, reinforcing the importance of integrated behavioral guidelines to prevent poor nutritional outcomes in youth [20].

Tan et al. (2024) [32] explored intergenerational nutritional problems in the Philippines, identifying the coexistence of maternal overweight and child stunting, illustrating how both under- and overnutrition can coexist within the same household, particularly under food-insecure conditions [32].

Furthermore, it is crucial to examine how factors like accessibility, cost, and food marketing strategies influence eating behavior and contribute to the increase in obesity and undernutrition [33]. Aggressive marketing of ultraprocessed foods, particularly in low-income communities, has led to an increase in the consumption of nutritionally poor food products, perpetuating harmful eating habits in the long term. This makes it more difficult to access nutritious and sustainable food options [34,35].

Moodie et al. (2013) [36] highlight the negative impact of food marketing, especially in vulnerable communities, where the promotion of ultraprocessed foods favors harmful food choices. These marketing practices not only increase the consumption of these products but also perpetuate unhealthy eating patterns, making it even harder to access healthier and more sustainable food options [36]. Stuckler and Nestle (2012) [37] discuss the influence of large food corporations, showing how their lobbying and marketing strategies shape global food systems and public health policies, creating an environment where harmful foods are more accessible and appealing, especially to the most vulnerable populations. These practices have long-term implications, as excessive consumption of ultraprocessed foods is directly linked to the rise of chronic diseases, such as obesity, diabetes, and cardiovascular diseases, while also exacerbating undernutrition due to the poor nutritional quality of these foods [37].

Poulsen et al. (2019) [38] corroborate this result, noting that BMI levels and body fat percentages are higher among youth from families severely affected by food insecurity or at risk of experiencing it [38]. Some studies explored the phenomenon of the double burden of undernutrition and obesity, identifying many children who suffer from hunger while coexisting with obese adults. These studies highlight the coexistence of stunting and obesity, suggesting the presence of hidden hunger [16,24].

Recent evidence from low- and middle-income countries reinforces this pattern. In Bangladesh, Gupta et al. (2023) [39] found significant rural-urban differences in the prevalence of underweight and overweight/obesity among adults, highlighting the coexistence of undernutrition and overnutrition within the same population. Their analysis of the Bangladesh Demographic and Health Survey 2017–2018 showed that underweight was more prevalent in rural areas, while overweight and obesity were more common in urban settings, underscoring the country’s double burden of malnutrition [39]. Similarly, in rural Central Java, Indonesia, Lowe et al. (2021) [21] observed dietary patterns characterized by low intake of fruits and vegetables and high intake of processed foods, which were associated with both undernutrition and overweight in the same population [21]. Finally, in Ethiopia, Dinku et al. (2020) [16] documented the simultaneous occurrence of childhood stunting and maternal obesity within households, illustrating the double burden of malnutrition in resource-limited settings [16].

Hidden hunger refers to a condition where individuals may be consuming enough calories, but their diets lack essential micronutrients, leading to deficiencies that are not immediately visible but have serious long-term health consequences. This form of hunger is often driven by the consumption of ultraprocessed foods, which are typically high in calories, sugars, and unhealthy fats, but low in essential nutrients. These foods are often more accessible and cheaper than healthier options, particularly in low-income communities, and their consumption can lead to an increase in BMI and the development of obesity, even though they do not meet the body’s nutritional needs. In essence, while individuals may appear to have an adequate caloric intake, their nutritional deficiencies remain hidden, contributing to the paradox of obesity and undernutrition coexisting [40]. Ortiz-Marrón et al. (2022) [23] emphasized that food insecurity is more prevalent in families with lower educational levels and purchasing power, associating it with inadequate eating habits and a higher prevalence of overweight and obesity [23]. Furthermore, Nel and Steyn (2022) [19] highlighted significant regional variations in diet and health conditions in Sub-Saharan Africa, while Keenan et al. (2021) [41] indicated that domestic food insecurity is related to increased BMI, mediated by stress and inadequate coping behaviors. Similarly, in the study by [25], it was observed that while women had a high prevalence of obesity (57.3%) and an average BMI of 26.21 kg/m², both men and women exhibited diets with low food diversity and nutritional deficiencies. The insufficient intake of essential foods, such as fruits and dairy products, coupled with the prevalence of chronic diseases, suggests that inadequate diets contribute to the coexistence of obesity and nutritional deficiencies, thus characterizing the double burden of malnutrition. This phenomenon underscores how undernutrition can persist even in contexts of food excess, often driven by the consumption of ultra-processed foods [25]. These results highlight the urgent need for integrated interventions that address the socioeconomic determinants of food insecurity and their consequences for nutritional health. Such interventions must consider factors such as educational level, socioeconomic status, the role and impact of the food industry, and access to healthy foods, aiming to promote healthy eating habits and combat both obesity and undernutrition. Moreover, it is crucial to implement more effective coping strategies to deal with the stress associated with food insecurity, as well as to promote educational programs that encourage the consumption of nutrient-rich foods tailored to the population’s nutritional needs, especially in contexts of social vulnerability. The development of public policies that integrate health, education, and social assistance is fundamental to reducing disparities in access to healthy food and, consequently, improving the quality of life and nutritional health of affected populations.

#### 4.1.2. Food Consumption, Obesity, and Climate Change

The relationship between food consumption, obesity, and climate change was explored in two articles [13,42]. Niles et al. (2021) [18] demonstrated that global food diversity among five-year-old children varies substantially due to climatic, agroecological, and socioeconomic factors such as climate vulnerability and the inequalities faced by marginalized populations [18]. These findings emphasize the role of climate change in exacerbating existing inequalities in food access and nutrition, particularly in vulnerable communities. Similarly, Owino et al. (2022) [8] discussed how climate change intensifies the disparities in food systems, affecting the availability of nutritious foods and leading to increased vulnerability to both obesity and undernutrition, especially among populations already facing socio-economic challenges [8].

The authors [18] highlighted that average food diversity is highest in South America (4.48) and lowest in Sub-Saharan Africa (2.66). Additionally, they observed that high temperatures are associated with a reduction in food diversity, while high precipitation correlates positively with diversity in some regions. A study [13] analyzed dietary changes in China between 1997 and 2011, identifying a decrease in the consumption of cereals and vegetables, accompanied by an increase in obesity and overweight, which grew from 26% to 44% during the studied period. The authors found a positive correlation (r = 0.92, *p* < 0.05) between obesity/overweight and the carbon footprint (CF) of foods, emphasizing that reducing beef consumption contributes to a lower carbon footprint and a reduced risk of obesity [13].

Although this topic was underexplored in this review, with only two articles, they indicated that development policies should integrate detailed climatic considerations to improve child nutrition, given the relationship between temperature variations, obesity, and food security. Evidence suggests that rising temperatures may impact BMI in children and adults, especially in developing countries, where vulnerability to climate change effects is higher [13], which aligns with the findings of this review.

Moreover, the climate’s impact on food security is more pronounced in tropical regions and economically disadvantaged populations, being exacerbated by high GHGE scenarios, which increase food prices and affect the availability of nutritious products [8]. This situation highlights the need for addressing climate and food justice, particularly by focusing on vulnerable populations that are disproportionately affected by the combined pressures of climate change and food insecurity [43]. Dietary interventions that encourage reducing the consumption of high environmental impact meats and promote plant-based diets can offer benefits for both health and the environment, reducing GHGE and decreasing obesity risks associated with poor dietary patterns [13]. Thus, the studies highlight that both climatic factors and food choices critically impact child nutrition and public health, underscoring the need for integrated approaches that consider these aspects in the formulation of policies and nutritional interventions [43].

#### 4.1.3. Food Consumption, Obesity, Undernutrition, and Climate Change

The research findings of Aceves-Martins et al. (2023) [17] provided valuable insights into the interrelationship between obesity, undernutrition, and climate change, considering socio-economic and demographic differences in the United Kingdom. The study indicated that participants with a BMI greater than 25 kg/m^2^ have a higher environmental food impact. This phenomenon reflects a diet that includes a higher proportion of high environmental impact foods, such as red meats, which are associated with higher costs and a larger carbon footprint. In contrast, the reduced calorie intake observed among women, whose diets are linked to lower values of a diet quality index (NRF8.3) and GHGE, may be influenced by societal beauty standards that prioritize thinness over health, encouraging restrictive, calorie-reduced diets that often exclude nutritious foods [17].

Furthermore, “social pressures” can be better understood through the lens of marketing campaigns targeting women, where processed and low-nutrient foods are frequently promoted as part of a healthy lifestyle. These campaigns, often backed by social media and influencer culture, encourage choices that do not prioritize nutritional value, thereby reinforcing unhealthy eating patterns. These dynamics highlight the intersectionality of gender and societal pressures in shaping eating habits, with women facing additional barriers due to cultural expectations, economic limitations, and the overwhelming influence of commercial marketing strategies [44,45].

The economic impact of these dietary choices also reflects the broader environmental and social consequences. As women, particularly in lower-income brackets, are pushed towards less expensive, calorie-dense foods, the link between food costs and climate change becomes evident. The preference for less sustainable, high-calorie, low-cost foods not only exacerbates the risk of malnutrition but also contributes to a higher carbon footprint, reinforcing the need for policies that address both the financial and environmental aspects of food choices [43].

This pattern is crucial for understanding climate change because less varied and sustainable diets may perpetuate a cycle of poverty and poor nutrition, exacerbated by adverse climatic conditions. Climate change, by affecting food availability and costs, intensifies these inequalities, making access to healthy and sustainable diets even more challenging for vulnerable populations. In an interview, Swinburn emphasized the central role of unhealthy foods in the global syndemic, particularly ultra-processed foods, which contribute to both obesity and undernutrition while driving negative environmental impacts [3]. The Lancet’s report on the global syndemic [3] underscores the need for dual and triple-action approaches that integrate environmental, food, and public health policies to simultaneously mitigate the impacts of these three interconnected pandemics.

The review of Dietz & Pryor (2022) [45] highlighted the need for behavioral and policy changes to address the global syndemic, particularly in the context of transportation and food production systems in the U.S. This research also reveals a lack of studies attempting to understand the issue of the global syndemic, as only one original study was identified in this review [46]. Despite this gap, the review by de Carvalho et al. (2024) [46], which aimed to understand the global syndemic through the lens of complex systems, proposed a systems dynamics model that maps the interactions between the components of the global syndemic in children under five, identifying structural factors and feedback loops that perpetuate these challenges and may contribute to the formulation of public policies. Thus, the observed differences between socio-economic groups in terms of nutritional quality, environmental impact, and food costs underscore the complex intersection of obesity, undernutrition, and climate change.

### 4.2. Conceptual Model

The model presented in Figure 4 offers a comprehensive and detailed view of the various factors influencing food security, highlighting the complexity of this issue. It helps us understand that food consumption is not solely determined by individual choices but by a set of interconnected factors, such as income, education, family structure, sanitation conditions, and even environmental changes. This approach allows us to view food security more realistically, considering not only the availability of food but also its accessibility, stability in supply, and how food is utilized in the diet.

Although the traditional pillars of food security address physical and economic aspects, the addition of agency expands the framework to include autonomy, voice, and participation in food system governance. The concept of agency refers to the capacity of individuals and groups to make informed decisions about what they eat, what food they produce, and how that food is distributed, regardless of external constraints [31]. In the context of the Global Syndemic, agency plays a vital role by enabling communities to resist structural determinants of poor nutrition and environmental degradation through informed, collective action [47].

The inclusion of environmental factors, such as agriculture and climate change, demonstrates that food security cannot be analyzed in isolation, as it is directly connected to issues such as temperature, precipitation, and GHGE. For example, climate variations can affect agricultural production, reducing the supply of certain foods and impacting their accessibility, particularly for more vulnerable populations.

Another essential aspect addressed in the model is food quality. It is not enough for food to be available; its diversity and nutritional value must also be considered. The model shows how inadequate dietary patterns can lead both to undernutrition and to an increase in chronic diseases, such as obesity and diabetes. This perspective broadens our understanding of the relationship between food and public health, reinforcing the importance of policies that promote balanced and sustainable diets.

Thus, by bringing together different factors in the same framework, the model helps us visualize the challenges of food security in an integrated manner. Its application can be crucial for the development of public policies and programs aimed at mitigating food insecurity, ensuring that the solutions adopted take into account the interdependence of various determinants of food and nutrition.

### 4.3. Countries Analyzed, Income Category According to the World Bank, and Relations with Food Security

Among the 62 countries studied in the titles included in this review (Supplementary Material Table S5), the majority were classified as low income (n = 22) and lower-middle income (n = 25) according to the Gross National Income (GNI) per capita of World Bank country groups and loans, which define economies as follows: low income (US$1145 or less in 2023); lower-middle income (GNI per capita between US$1146 and US$4515); upper-middle income (GNI per capita between US$4516 and US$14,005); high income (GNI per capita above US$14,005) [48].

This classification reflects the direct consequences found between income and food security. The highest levels of food insecurity are found in countries where the population has lower purchasing power. Among the locations studied in this review, in 2023, Sub-Saharan Africa had the highest moderate and severe food insecurity index (63.3%), followed by North Africa (33.8%); Central America (28.2%); South America (25.1%); Asia (24.8%); and Europe (8.2%) [5].

Notably, even in high-income countries [21,23,34], socioeconomic disparities negatively affect dietary behaviors, diet quality, and food security among marginalized populations.

Dietary patterns driven by the hegemonic food system not only reflect income-based disparities in food security levels but also shape broader eating habits. This dominant production and consumption model permeates food culture and undermines food sovereignty: diets grow increasingly monotonous and ultraprocessed food-laden—purchased either in supermarkets or fast-food chains—across both high- and low-income populations [49]. Consequently, communities progressively lose agency over how to produce, process, distribute, access, and consume nutritious foods, regardless of race, social class, gender, ethnicity, or religion [50,51].

This reality necessitates critical examination of prevailing food-related cultural norms to revitalize traditional dietary patterns, which are associated with healthier diets and inherently promote more sustainable food systems. Such systems emphasize agricultural biodiversity and equitable food production/consumption [2]. Achieving this transformation requires coordinated efforts between civil society and policymakers to counteract commercial pressures from multinational corporations—key drivers of the dual malnutrition/obesity pandemic [49] and climate change exacerbation—while implementing public policies that simultaneously improve food security metrics and advance Sustainable Development Goal (SDG) targets.

### 4.4. Limitations and Contributions of the Study

While this study offers important contributions, it is essential to recognize some limitations. During the scoping review, it became evident that there is a lack of research investigating, in an integrated manner, the relationship between malnutrition, climate change, and food consumption. Furthermore, we observed the absence of studies addressing these issues in populations from Latin America, which hinders the direct application of the results to our context. A limitation was also noted in the number of studies that consider all three components of the global syndemic—undernutrition, obesity, and climate change—in conjunction with food consumption. Only one of the articles included in the review addressed this intersection in a more comprehensive way.

Despite these limitations, this study presents significant strengths. The scoping review was crucial in identifying gaps in the literature, allowing future research to be directed at filling these gaps and deepening knowledge in the field. Furthermore, the development of the conceptual model presented in this work represents a relevant contribution, as it organizes and connects the various factors influencing food security.

This model can serve as a useful tool both to guide new investigations and to support public policies aimed at finding more effective solutions to the challenges of food, nutrition, and climate change—as advocated by initiatives such as the Initiative on Climate Action and Nutrition (ICAN), which promotes integrated approaches to tackle malnutrition and environmental degradation simultaneously [5]. Thus, despite the limitations, the findings of this study emphasize the importance of the topic and point to promising avenues for future research and interventions.

## 5. Conclusions

This study explored the interconnection between food consumption, obesity, undernutrition, and climate change, highlighting the complexity and interdependence of these factors in the global context. From the literature review, it became clear that these issues should not be analyzed in isolation, as their relationships are multiple and dynamic, especially in regions that are more vulnerable from a socioeconomic and environmental standpoint. The conceptual model presented in this study offers an integrated view of food security, providing a better understanding of how factors such as food access, nutritional quality, social conditions, and environmental impacts intertwine.

The reviewed evidence shows that food insecurity, obesity, and undernutrition are not isolated phenomena but components of a global syndemic that disproportionately affects the most vulnerable populations. The relationship between these factors is particularly evident in regions with low income levels and high social inequality, where adverse climate conditions, such as temperature and precipitation variations, further exacerbate food availability and quality. Moreover, the growing prevalence of diets based on ultra-processed foods is directly linked to the rising rates of obesity, intensifying the cycle of food insecurity and nutritional poverty.

Therefore, it is essential that public policies take an integrated approach to food security, environmental sustainability, and health promotion. Interdisciplinary strategies that promote balanced and sustainable diets and ensure access to nutritious foods are key to mitigating the negative effects of this global crisis. To achieve this, future studies should focus on more detailed models, considering regional specificities and developing interventions based on concrete, context-driven data.

In summary, this study reaffirms the need for an integrated approach to address the crisis involving obesity, undernutrition, and climate change. This crisis primarily affects the most vulnerable populations, and ongoing research in this area will be crucial for developing more effective and adaptable solutions that improve living conditions and health, particularly for those in situations of greater economic and environmental vulnerability.

## Data Availability

The data supporting the findings of this study are available within the article and its Supplementary Materials.

## Supplementary Materials

The following supporting information can be downloaded at: https://www.mdpi.com/article/10.3390/ijerph22060897/s1, Table S1. Search Strategy—SCOPUS via Elsevier (Search conducted on 11 March 2024); Table S2. Search Strategy—MEDLINE/PubMed (via National Library of Medicine) (Search conducted on 11 March 2024); Table S3. Search Strategy—Web of Science—Core Collection (Clarivate Analytics) (Search conducted on 11 March 2024); Table S4. Data Extraction Instrument; Table S5. Countries Present in the Articles Included in the Review and Classification According to Income (GNI) per Capita by World Bank Country Groups and Loans; Table S6. Expert Panel Decisions on the Connections Between the Causes and Consequences of the Global Syndemic; Table S7. Characteristics of Selected Studies and Global Syndemic Components and Code Management in the Review; Table S8. Frequency of Occurrence of the Variable in the Review.
